# mTORC1 Is Not Principally Involved in the Induction of Human Endotoxin Tolerance

**DOI:** 10.3389/fimmu.2020.01515

**Published:** 2020-08-07

**Authors:** Kristin Ludwig, Ralf A. Husain, Ignacio Rubio

**Affiliations:** ^1^Institute of Molecular Cell Biology, Center for Molecular Biomedicine, University Hospital Jena, Jena, Germany; ^2^Department of Neuropediatrics, University Hospital Jena, Jena, Germany; ^3^Clinic of Anaesthesiology and Intensive Care and Center for Sepsis Control and Care (CSCC), University Hospital Jena, Jena, Germany

**Keywords:** mTORC1, endotoxin tolerance, monocytes, macrophage, sepsis, immune suppression

## Abstract

Endotoxin tolerance represents a safeguard mechanism for preventing detrimental prolonged inflammation and exaggerated immune/inflammatory responses from innate immune cells to recurrent harmless pathogens. On the other hand, excessive immune tolerance can contribute to pathological immunosuppression, e.g., as present in sepsis. Monocyte activation is accompanied by intracellular metabolic rearrangements that are reportedly orchestrated by the metabolic signaling node mTORC1. mTORC1-dependent metabolic re-wiring plays a major role in monocyte/macrophage polarization, but whether mTORC1 participates in the induction of endotoxin tolerance and other immune adaptive programs, such as immune training, is not clear. This connection has been difficult to test in the past due to the lack of appropriate models of human endotoxin tolerance allowing for the genetic manipulation of mTORC1. We have addressed this shortcoming by investigating monocytes from tuberous sclerosis (TSC) patients that feature a functional loss of the tumor suppressor TSC1/2 and a concomitant hyperactivation of mTORC1. Subjecting these cells to various protocols of immune priming and adaptation showed that the TSC monocytes are not compromised in the induction of tolerance. Analogously, we find that pharmacological mTORC1 inhibition does not prevent endotoxin tolerance induction in human monocytes. Interestingly, neither manipulation affected the capacity of activated monocytes to switch to increased lactic fermentation. In sum, our findings document that mTORC1 is unlikely to be involved in the induction of endotoxin tolerance in human monocytes and argue against a causal link between an mTORC1-dependent metabolic switch and the induction of immune tolerance.

## Introduction

Innate immune cells of the myeloblastic lineage constitute the first line of defense against infection and tissue breakdown in trauma. Upon identifying, spotting, tracking, or engulfing pathogens, pathogen-associated molecular patterns (PAMPs), or damage-associated molecular patterns, the myeloblastic cells elicit a cascade of inflammatory and immune responses mediated by the release of cytokine cocktails and eventually the direct presentation of antigen to lymphocytes. Due to their unique ability to recognize and rank infectious or traumatic triggers, the innate immune cells dictate the quality and the intensity of the host response and hence the course of an infection episode. Owing to this primordial role at the vanguard of the host response, the innate immune cells possess intricate mechanisms for fine-tuning their immune responses according to the risk and the severity of any particular infection. One level of control is provided by the limited lifespan of monocytes or neutrophils (as new immunocytes continuously emerge from the bone marrow), which precludes the pernicious accumulation of hyper-reactive or aberrant immune cells. Additional fine-tuning proceeds at the molecular level as the innate immune cells are able to adapt dynamically to a particular infection and trauma scenarios and re-shape their response accordingly. For example, in a process known as immune training, the activated monocytes re-configure their response toward ensuing inflammatory cues in the long term *via* PAMP-induced changes in the epigenome (1, 2).

Another important process that can shape the amplitude and the quality of monocyte responses is endotoxin tolerance (ET) (3). ET represents a well-established state of hyporesponsiveness characterized by a skewed, largely anti-inflammatory response, which is intended to prevent exaggerated immune/inflammatory responses to recurrent and innocuous antigens. Several models have been put forward to explain tolerance induction in monocytes, but the precise molecular mechanisms remain elusive (3, 4). Besides the intellectual challenge of deciphering the molecular processes that mediate ET in the monocyte, understanding the tolerance mechanisms is relevant also from a clinical perspective because untimely or unleashed tolerance contributes to states of immunosuppression in lethal conditions such as sepsis (5, 6).

Most models of endotoxin tolerance invoke molecular rearrangements downstream of TLR4 or other pattern recognition receptors (PRR) in the tolerant immune cell (4, 7). Thus, up-regulation/activation of the downstream kinase IRAK-M has been shown to block TLR4 signaling at the level of Myd88-containing complex (Myddosome) formation, promoting a state of tolerance toward lipopolysaccharide (LPS) (8, 9). Another focus has been placed on the characterization of feedback mechanisms that attenuate signaling by PRRs in tolerized cells. Many of those feedback models invoke autocrine loops, including the secretion of anti-inflammatory cytokines such as IL-10 or TGFß that can, moreover, contribute to a generalized environment of immune suppression by impinging on other immune cells such as lymphocytes (10, 11). Indeed the endotoxin tolerance of circulating monocytes from sepsis patients correlates with high levels of IL-10 and other anti-inflammatory cytokines and is characterized by high intramonocytic levels of IRAK-M protein (8). Despite these advances in our understanding of endotoxin tolerance, however, a unifying model of tolerance induction is still lacking as several important features of ET in monocytes remain unaccounted for.

One such poorly understood aspect that has raised much interest is with regards the immunocyte's metabolism and the potential role of metabolic re-wiring processes in the course of ET induction. Recently, a metabolic switch toward anaerobic glycolysis, analogous to the Warburg effect originally described for cancer cells, has been put forward as a crucial step for immune training of monocytes (12, 13). Indeed as documented in multiple reports dating back to the 1960s, monocytes experience a pronounced Warburg-like metabolic switch upon immune activation, leading to more aerobic glucose fermentation and lactate production with a concomitant drop in cellular respiration (14, 15). It is generally assumed that these metabolic rearrangements serve the purpose of optimizing energy production and expenditure, arming and preparing the monocyte to combat the infectious threat in the inflamed tissue. However, whether or not Warburg-like metabolic rearrangements are an integral component of immune adaptive programs leading to ET or immune training is not clear.

The metabolic switch in immunocytes and other cell types is presumably orchestrated by a limited number of cellular master metabolic regulatory proteins, prominently the energy and nutrient sensors AMPK, HIF1α, and mTORC1. The metabolic signaling node mTOR containing complex 1 (mTORC1) is a multi-protein complex named after its core component, the Ser/Thr kinase mTOR. It acts as a master intracellular hub of metabolic control as it funnels and records hormonal, environmental, and intracellular cues reporting nutrient and energy availability [reviewed in (16)]. mTORC1 processes and converts this information into an appropriate signaling output that orchestrates catabolic and anabolic processes in the cell. Mechanistically, mTORC1 activity is controlled by the action of tuberous sclerosis 1 and 2 (TSC1/TSC2) tumor suppressor protein complex, an immediate upstream negative regulator acting as a GTPase-activating protein for the small G-protein Rheb (17, 18). mTORC1 activity is critical for immune cell function as the pharmacological inhibition of mTORC1 can substantially alter or, in some cases such as T-lymphocytes, completely ablate immune responses (19). Indeed the mTORC1 inhibitor rapamycin and its derivatives, like everolimus, are immunosuppressants commonly used in the clinic. Thus, while it is firmly established that mTORC1 activity is critical for immune cell function, it is not known whether it plays a direct role in adaptive processes such as ET. Such a connection has proved to be difficult to test experimentally, other than by using pharmacological inhibitors like rapamycin. However, pharmacological approaches suffer from a number of drawbacks and thus need to be complemented by genetic approaches, which are difficult to implement on primary immune cells.

To circumvent these methodological shortcuts, we have investigated monocytes from TSC patients that feature a functional loss of TSC1/2 and a concomitant hyperactivation of mTORC1. TSC is an autosomal dominant disorder caused by loss-of-function germ-line variants of either of the two TSC protein complex components TSC1 and TSC2 (20, 21). TSC1 and TSC2 together form the upstream negative regulator TSC complex for mTORC1. TSC patients manifest multiple benign neoplasias, designated as hamartomas, that can affect many organs and are often characterized by exorbitantly large, giant cells. Although TSC1 and/or TSC2 have been attributed additional functions beyond acting as a gatekeeper for mTORC1 (22), it is generally accepted that the clinical manifestations of TSC result principally from hyperactive mTORC1 signaling. Thus, TSC represents a “genetic model” for mTORC1 gain-of-function. Here we subjected monocytes from TSC patients to various protocols of immune adaptation to test if they were compromised in the induction of tolerance or training. As presented and discussed below, our findings strongly argue against a role for mTORC1 in the induction of immune tolerance.

## Materials and Methods

### TSC Patient Enrolment and Ethics

Patient enrolment and blood drawing were performed at the Department of Neuropediatrics, University Hospital Jena, Germany. The study was approved by the local ethics committee of the University Hospital Jena (study registry number: 4498-07/15). Written informed consent was obtained from all the study participants or their legal representatives before the blood drawing. All patients included were diagnosed with TSC on the basis of gene sequencing (16 out of 19 patients) and/or unambiguous clinical features of TSC (Table 1). The exclusion criteria included recent/acute episodes of inflammation or infection, a CrP value >10 mg/l, any type of chronic disease, and treatment with immunosuppressive other than everolimus at the time point of blood drawing. Further patient and healthy donor characteristics are listed in Table 2.

**Table 1:** Spectrum of the genetic lesions mapped to TSC1/TSC2 and the clinical features of the patients enrolled in the study.

**Internal subject #**	**Clinical features (tuberous sclerosis specific)**	**Genetic mutation**
002	SEN, SEGA, EPI, ID, RAML, HM, FA	TSC2: c.5135C>T
003	SEN, CD, EPI, DD, RAML, RC	TSC2: c.2251C>T
005	SEN, CD, SEGA, EPI, ID, RAML, RC, CR, AR, HM, FA	TSC2: c.5110del
008	SEN, CD, SEGA, EPI, ID, RC, CR, HM, FA	TSC2: c.1287dup
009	SEN, CD, EPI, RAML, RC, CR, MMPH, HM, FA	TSC2: c.976-15G>A
010	EPI, FA	TSC1: c.211-1G>A
011	SEGA, EPI, ID, RC, CR, FA	TSC2: c.? (written report not available)
012	EPI, RC, CR, HM, FA	TSC2: deletion exons 30-41
013	EPI, RC, FA	Not available
016	SEN, CD, DD, RC, CR, AR, HM	TSC2: deletion exons 15-21
017	SEN, CD, EPI,DD, HM, FA	TSC2: c.1832G>A
018	SEN, CD, SEGA, EPI, ID, RAML, RC, CR, AR, HM, FA	TSC2: c.5110del
020	SEN, CD, SEGA, EPI, ID, RC, CR, HM	TSC2: c.4925G>A
021	SEN, CD, SEGA, EPI, ID, RAML, CR, HM, FA	TSC2: c.? (written report not available)
024	SEN, CD, SEGA, EPI, ID, RAML, RC, CR, HM, FA	TSC1: c.2029insC
026	SEN, CD, SEGA, EPI, ID, RAML, RC, HM, FA	TSC2: c.4646A>G
029	SEN, CD, EPI, DD, HM	TSC2: c.4712A>G
031	SEN, CD, EPI, ID, PI, RAML, RC, CR, HM, FA	TSC2: c.1832G>A
036	SEN, CD, RC, CR	TSC2: c.3284+1G>A

*SEN, subependymal nodules; SEGA, subependymal giant cell astrocytomas; EPI, epilepsy; ID, intellectual disability; RAML, renal angiomyolipomas; HM, hypomelanotic macules; FA, facial angiofibromas; CD: cortical dysplasias; DD, developmental delay; RC, renal cysts; CR, cardiac rhabdomyomas; AR, arrhythmias; PI, psychiatric illness*.

**Table 2:** Patient and healthy donor characteristics.

**Characteristics**	**Number**
**Tuberous sclerosis patients**
Total	19
Male	13
Female	6
Age (mean/median)	12/12
Age range (years)	0–38
Everolimus treatment	7
TSC1 mutation	2
TSC2 mutation	14
Mutation unknown	3
**Healthy donors**	
Total	25
Male	16
Female	9
Age (mean/median)	13/13
Age range	0–42

### Materials

Rapamycine was from Calbiochem. Torin-1 was from TOCRIS. LPS (strain055:B5) was purchased from Sigma-Aldrich (#L2880). ß-Glucan was obtained from two sources: (1) a kind gift of Mihai Netea, Nijmegen, Netherlands and (2) a kind gift of David L. Williams, Johnson City, USA.

The proteome profiler Human Cytokine Array Kit was from R&D Systems; the cytometric-based bead array (CBA) flex sets for multiplexed cytokine determinations were acquired from BD Biosciences. Ficoll Histopaque®-1077 was from Sigma-Aldrich. The ELISA-standard TNFα was from Biolegend, Inc.

### Antibodies

The antibodies for western blotting, S6-Protein (5G109), p-S6-Protein (Ser235/236), AKT, p-AKT (D9E) (Ser473), ERK1/2 (137F5), and p-ERK1/2 (E10) (Thr202/204), were all purchased from Cell Signaling. Anti-p38 and p-p38 (Thr108/Tyr182) were from BD Transduction. All antibodies were used at 1:1,000 dilution in TBS-Tween supplemented with 1% BSA. The antibody for flow cytometry was AntiCD14 (Immunotools).

### Monocyte Isolation and Cultivation

Blood was drawn using Li/heparin monovettes by trained physicians. EDTA-blood from patients and control donors was blinded on-site at the neuropediatrics unit, transported at room temperature to the laboratory within <4 h of drawing, and processed immediately. Peripheral blood mononuclear cells (PBMCs) were isolated by standard density gradient centrifugation on Ficoll. Briefly, blood was diluted with isolation buffer [phosphate-buffered saline (PBS) without Ca^2+^/Mg^2+^, 1% BSA, 2 mM EDTA] to a final volume of 30 ml. The blood–buffer solution was carefully layered on 15 ml Ficoll Histopaque®-1077 solution and centrifuged at 800 g for 20 min (without break). The PBMC layer was harvested and washed twice with cold isolation buffer. The cells were resuspended in RPMI 1640 medium supplemented with 10 μg/ml gentamycine, 1% sodium pyruvate, 1% GlutaMax, and 10% heat-inactivated human serum (Sigma-Aldrich) and seeded at a density of 5–10 × 10^6^ cells/ml. The monocytes were further purified on the basis of differential attachment to cell culture dish surfaces. The cells were left to settle and attach to the culture plate surface for 1 h at 37°C. The non-adherent cells representing non-monocytic fractions were washed off by three rounds of mild rinsing with warm PBS without Ca^2+^/Mg^2+^. The purity of the monocyte preparations was assessed by flow cytometry staining for surface CD14. Purity was routinely 90% or higher. The test runs of monocyte preparations using magnetic anti-CD14 beads yielded virtually identical purity and undistinguishable experimental results (not shown). The monocytes were cultured at 37°C and 5% CO_2_ in a humidified atmosphere.

### Monocyte Priming and Stimulation

Priming (either tolerance induction by LPS or training by ß-glucan) was performed by the treatment of monocyte cultures with 10–100 μg/ml LPS or with 3 μg/ml β-glucan for 24 h, respectively. At the end of the priming period, the cells were washed three times by rinsing with warm medium and subsequently stimulated with fresh medium containing 10 ng/ml LPS for additional 24 h. The cell culture supernatants were collected, cleared from cell debris by centrifugation (10,000 g for 10 min), and analyzed for cytokine production and metabolic parameters or stored at −20°C until analysis. The cells were collected by mild centrifugation (600 g for 10 min at 4°C) and analyzed by flow cytometry as appropriate. For inhibition of mTORC1, the cells were pre-incubated with a mixture of 20 ng/ml rapamycin and 10 ng/ml Torin-1 for 30 min prior to priming or stimulation with PAMPs. In the primed cells, both inhibitors were present during the 24-h period of priming.

### Cytokine Profiling by Cytokine Strips/Proteome Profiler Human Cytokine Array

The cell culture supernatants were collected at the indicated time points, cleared from cell debris, and stored at −20°C until analysis. The cytokine profiles were determined using cytokine strips (R&D Systems) according to the manufacturer's instructions. The signals were detected by exposure to X-ray films and quantitated/imaged on a LAS system.

### Cytokine Profiling by ELISA

TNFα production was measured in cleared cell culture supernatants by ELISA in accordance to the manufacturer's protocol. Colorimetric detection was performed with 3,3',5,5'-tetramethylbenzidine substrate solution (Biolegend, San Diego, CA, USA), and the reactions were quenched by the addition of 2N H_2_SO_4_. Absorbance was measured at 450 nm with a TECAN Microplate Reader (VersaMax) and analyzed using SOFTmax Pro software (Molecular Devices).

### Cytokine Profiling by Multiplexed Bead Arrays/Cytometric Bead Array Flex Set

The production of nine defined inflammatory cytokines in cleared cell culture supernatants was measured by flow CBA on a FACSCanto™II, following the manufacturer's instructions. Data analysis was carried out using Flow Jo software (TreeStar Inc.). The calculated cytokine amounts were normalized to protein content (determined on the pelleted cells measured using Pierce® Micro BCA Protein Assay Kit) to account for different viability or growth patterns under the various treatment conditions (23). All cytokine concentrations are plotted as normalized cytokine amount per milliliter of supernatant.

### Western Blotting

Human monocytes were seeded on six-well plates at a density of 10^7^ cells/ml and left to attach for 1 h at 37°C, followed by three rounds of gentle washing to remove non-adherent cells. The cells were either primed with LPS or β-glucan in the presence or the absence of mTOR inhibitors or left untreated for 24 h. After priming, the cells were washed three times with medium, followed by stimulation with 10 ng/ml LPS for 30 or 60 min. The reactions were quenched with ice-cold RIPA lysis buffer (50 mM HEPES pH 7.5, 150 mM NaCl, 5 mM EDTA, 1% NP-40, 0.5% deoxycholate, and 0.1% SDS, supplemented with protease inhibitors) and the cell extracts were cleared by centrifugation. The protein concentration was determined with the BCA Protein Assay. The samples were treated with Laemmli buffer, boiled for 5 min, and equal amounts of total protein were resolved by SDS-PAGE. The proteins were transferred to polyvinylidene difluoride membranes using Trans-Blot Cell Tank system (Bio-Rad^TM^) for wet blotting and probed with the indicated antibodies. The signals were quantified by densitometry on a ImageQuant™ LAS 4,000 instrument.

### Stimulation With Conditioned Medium

Conditioned media were collected from human monocyte cultures as follows: the cells grown in full culture medium were challenged with 100 ng/ml LPS; at 1 h later, the cells were washed once and the medium was replaced with a fresh one without LPS. At 23 h later, the medium was collected, cleared from cell debris by centrifugation, and used immediately without intermediate storage as conditioned medium for the priming of naïve monocytes.

### Lactate and Glucose Measurements

The lactate and glucose levels from monocyte culture supernatants were measured by the in-house clinical chemistry department of the Jena University Hospital.

### Statistical Analysis

GraphPad Prism five and six were used for statistical analysis. All data are expressed as means ± SEM. A Wilcoxon matched-pairs signed rank test was performed to determine the significance between different treatments within one experimental group. Two-way ANOVA with Bonferroni post-test was used to determine the significance between two experimental groups (^*^p ≤ 0.05, ^**^p ≤ 0.01, ^***^p ≤ 0.001, ^****^p ≤ 0.0001). Different significance symbols were used to mark different inter-group comparisons.

## Results

### TSC Monocytes Feature Normal Response to LPS Challenge

**Figure 1:**
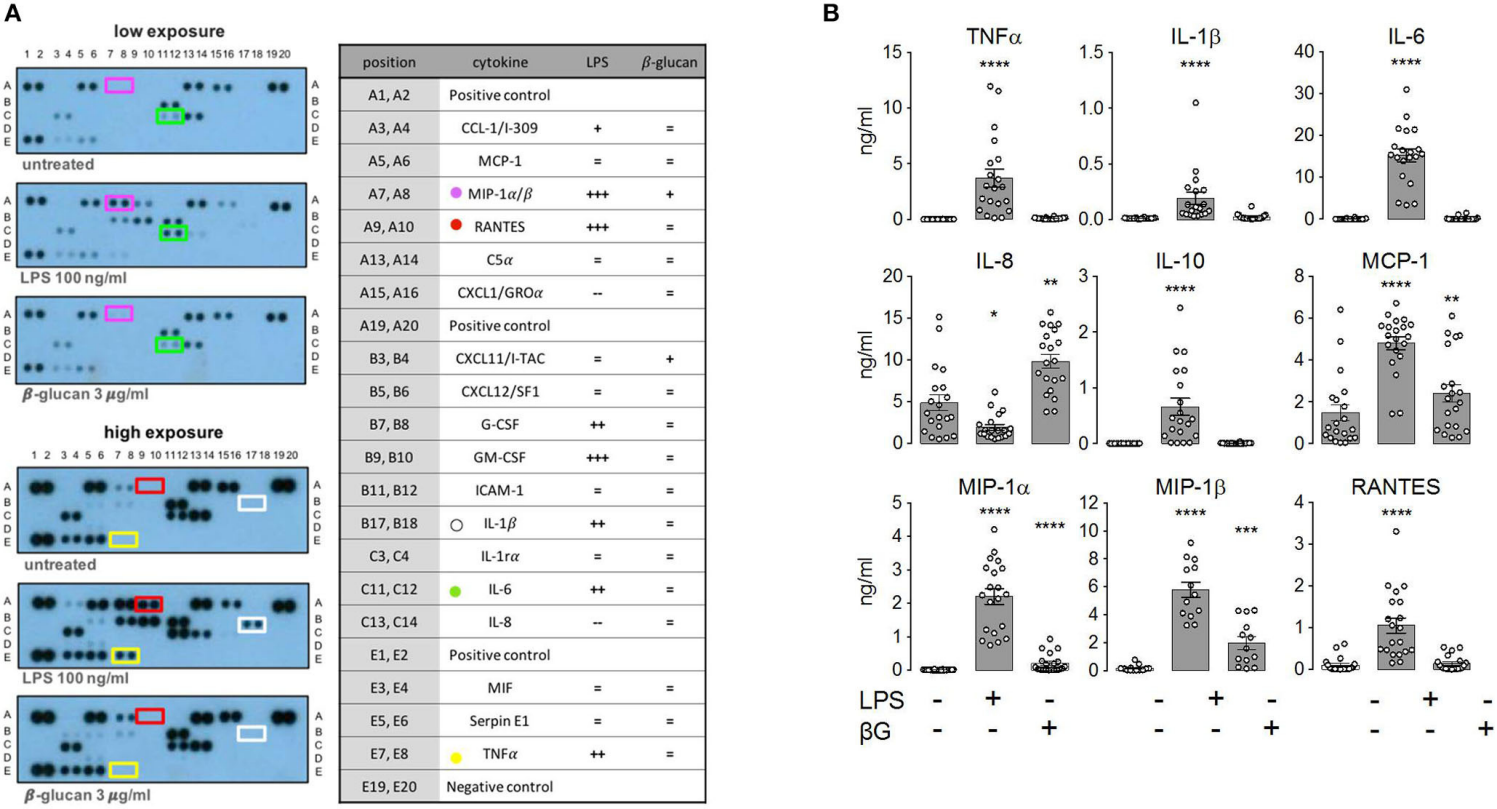
Lipopolysaccharide (LPS) and ß-glucan (ßG) exert distinct patterns of monocytic cytokine secretion. **(A)** Human peripheral monocytes from healthy volunteers were isolated, cultured, and stimulated with 10 ng/ml LPS or 3 μg/ml ß-glucan as described in the experimental section. At 24 h later, cytokine production was assessed with a human cytokine array encompassing 36 cytokines. The selected prototypical cytokines are highlighted with a color code. Changes in all 36 cytokines were scored and plotted in categories from strong down-regulation to strong up-regulation (—, –, -, =, +, ++, and +++). **(B)** Human monocytes from healthy donors were isolated and treated as before. The cytokine levels were assessed by ELISA at 24 h post-stimulation. Different significance symbols were used to mark different inter-group comparisons.

In order to test the role of mTORC1 on the plasticity and the adaptation properties of human monocytes, we investigated, side by side, monocytes from TSC patients and healthy donors. To this end, we collected blood from mostly infant TSC patients that visited the neuropediatrics department for a routine medical check. Whenever possible, blood from age-matched healthy donors was collected and assayed on the same occasion. In order to avoid interference with the immunological parameters under investigation, the exclusion criteria for TSC patients included immunosuppressive therapies or recent infectious episodes, among others (see section Materials and Methods). Seven out of the 19 enrolled patients received everolimus therapy at the time point of blood withdrawal. Nine patients donated blood twice, with a gap of 6 months or more in between, but the obtained values were treated as individual data sets. Monocytes were isolated by differential plate attachment/washout protocols or magnetic isolation based on the surface expression of CD14. The cells isolated by either protocol showed undistinguishable results (data no shown). We did not observe any obvious phenotypic differences between control and TSC monocytes during routine cultivation. To assess general monocyte responsiveness, we challenged the cells with the PAMP LPS, a cell wall constituent of gram negatives and a strong inducer of ET (2). In parallel samples we stimulated also with ß-glucan from *Candida albicans* as a PAMP that reportedly induces immune training in these cells (24). In order to obtain a broad view of the cytokine spectrum induced by both PAMPs, we first challenged the control monocytes from healthy donors for 24 h and loaded the supernatant on cytokine strips (Figure 1A). As reported before (25), LPS induced the secretion of multiple cytokines, including TNFα, IL-1ß, IL-6, RANTES, and MIP1, while it reduced the secretion of others, prominently IL-8. By contrast, ß-glucan was a poor secretagogue, causing the mild upregulation of but a few cytokines, at least as measured under these conditions. To exclude a lack of activity of the employed ß-glucan, we tested two ß-glucan preparations of different origins (see the experimental section). Both batches yielded undistinguishable results. A flow cytometric bead array-based assay, which produced better quantifiable results, confirmed the marked difference in the cytokine release proficiency of LPS vs. that of ß-glucan, the latter inducing only a modest secretion of IL-8, MIP-1, and MCP-1 (Figure 1B).

**Figure 2:**
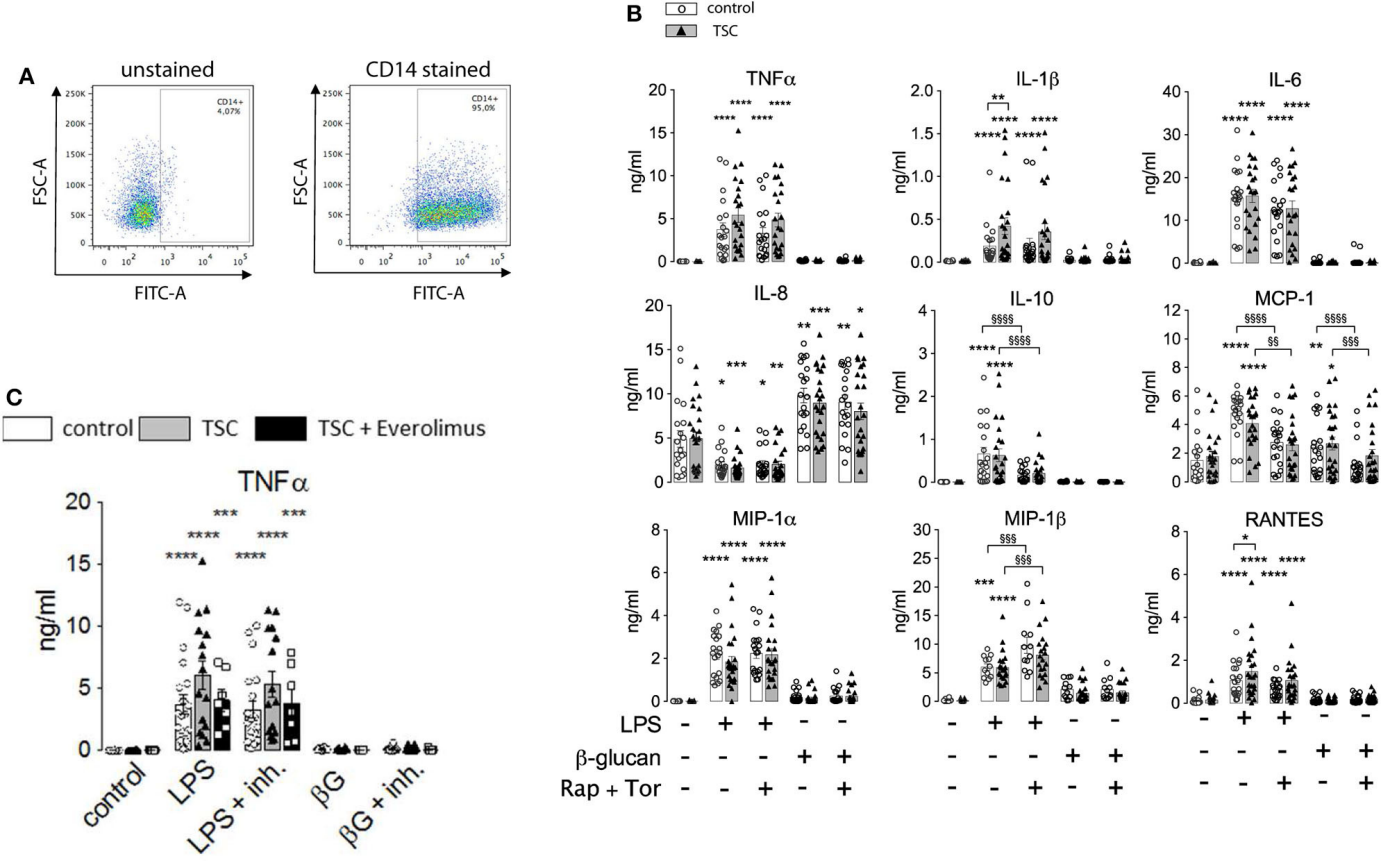
Monocytes from tuberous sclerosis (TSC) patients feature a largely unperturbed cytokine response to lipopolysaccharide (LPS) and ß-glucan. **(A)** Purity of human monocyte preparations from TSC patients as assessed by CD14 surface staining. The identified peripheral blood mononuclear cells were pre-gated for cellularity and doublet exclusion, followed by surface staining with or without anti-CD14 Abs. **(B)** Human peripheral monocytes from TSC and control groups were treated as indicated with a mix of rapamycin (Rap) and Torin1 (Tor) at 30 min prior to stimulation with 10 ng/ml LPS or 3 μg/ml ß-glucan. The cytokine levels in the supernatant were determined by flow cytometry using a multiplex bead array. **(C)** The same data from TNFα panel in **(B)** stratified for +/– everolimus treatment. Different significance symbols were used to mark different inter-group comparisons.

To assess the role of mTORC1, we performed side-by-side measurements on control and TSC monocytes (Figure 2). The purity of the monocyte preparations routinely exceeded 90%, as assessed by flow cytometry (Figure 2A). These experiments evidenced that monocytes from TSC patients were not markedly affected in their cytokine response to LPS (Figure 2B). In line with previous findings in mouse monocytes (26), some pro-inflammatory mediators were released even more profusely by the stimulated TSC monocytes, perhaps reflecting a generalized higher protein translation rate as a consequence of hyperactive mTORC1 signaling. In our experiments, this was true for TNFα, IL-1ß, and RANTES, achieving statistical significance for the latter two mediators. Seven out of the 19 TSC patients included in our study received everolimus therapy at the time point of blood drawing. Stratification of the data with regard to everolimus therapy showed that the higher cytokine release resulted in its majority from patients that had not received therapy with the mTORC1 inhibitor, suggesting an association between chronic aberrantly high mTORC1 signaling and enhanced cytokine release (Figure 2C). To test if cytokine production was affected by acute mTORC1 inhibition, we administered a combination of two potent mTORC1 inhibitors acting by different mechanisms: the allosteric inhibitor rapamycin and the ATP-competitive drug Torin-1. We used this inhibitor mix because rapamycin reportedly shows a selective inhibition of distinct mTORC1 downstream targets under particular conditions (27). Both inhibitors were administered simultaneously at 30 min prior to the stimulation with PAMPs. As can be seen in Figure 2B, mTORC1 inhibition prevented the production of IL-10 and MCP1, whereas the production of MIP-1ß was mildly enhanced in mTORC1-inhibited cells. The levels of all other cytokines were largely unaffected. The elevated production of TNFα, IL-1, or RANTES in TSC monocytes was largely attributable to the patients who were not treated with everolimus (Figure 2C; data not shown). Intriguingly, this elevated cytokine response in TSC cells was not prevented by inhibitor treatment, suggesting that acute mTORC1 inhibition could not revert the effect of chronic mTORC1 upregulation. In summary, the generation of individual cytokines by human monocytes was differentially dependent on mTORC1 but was not significantly compromised by the presence of unleashed mTORC1 activity in TSC.

### ET Proceeds Normally in TSC Monocytes

**Figure 3:**
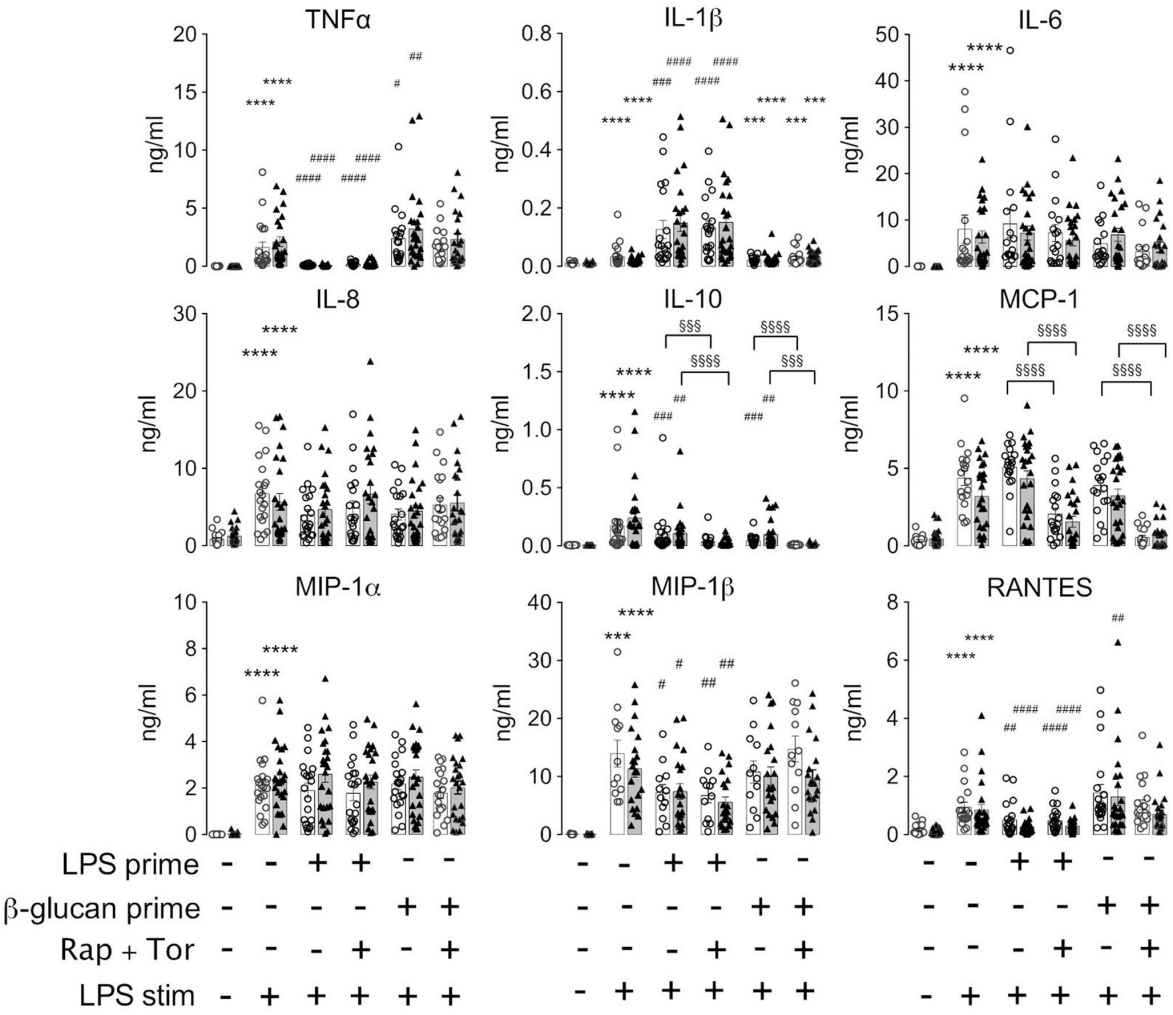
Monocytes from tuberous sclerosis (TSC) patients can be rendered tolerant by endotoxin. Peripheral monocytes from TSC patients (black triangles) or control healthy donors (white circles) were isolated and subjected to a bi-phasic priming/stimulation protocol for induction of endotoxin tolerance. The cells were primed with LPS or ß-glucan for 24 h (prime), followed by stimulation with 10 ng/ml LPS (stim) for 24 h further. The cytokine levels were measured by flow cytometric multiplexed bead arrays as before. Different significance symbols were used to mark different inter-group comparisons.

The above findings evidenced that the generation of cytokines was distinctively sensitive to chronic or acute changes in mTORC1 activity, as implemented by the TSC genotype and pharmacological mTORC1 inhibition. We went one step further and assessed whether mTORC1 played a role in the induction of ET. For this purpose, we altered the experimental protocol to include a priming step with LPS or ß-glucan. Whereas, priming with LPS for as short as 1 day is known to induce a state of tolerance in mouse macrophages and human monocytes, ß-glucan induces immune training in these cells (2, 24). At 24 h after the priming step, the cells were challenged with LPS and the cytokine levels were monitored 24 h later *via* flow cytometry on bead arrays. It is important to note that re-stimulation was accompanied by the replacement of the culture medium. This step largely removed all cytokines produced upon the initial LPS/ß-glucan priming step, as ascertained in control experiments (data not shown), excluding an adulteration of the measured cytokine levels. As shown in Figure 3, priming with LPS fully prevented the production of TNFα and partially suppressed that of RANTES, IL-10, and MIP-1ß. By contrast, LPS priming did not reduce the production of other cytokines, including IL-6, IL-8, or MCP-1, and even boosted the release of IL-1. Priming with ß-glucan for 24 h exerted only a little effect on the cytokine levels. Prolonging the priming step of ß-glucan to 5 days neither lead to training effects as those reported previously (24, 28). Importantly, the loss of TSC had no impact on ET induction as priming with LPS induced a largely undistinguishable re-wiring of cytokine production in control and TSC cells. These findings showed that mTORC1 hyperactivation, as present in TSC cells, did not prevent nor affect the molecular processes underlying the induction of ET in human monocytes. In line with a negligible role of mTORC1, the pharmacological inhibition of mTORC1 prior to and throughout the priming period did not also affect ET. Taken together, these data strongly suggested that mTORC1 activity or changes in its activity are not principally involved in the induction of ET in monocytes.

### Metabolic Switch Is Not Affected in TSC Monocytes and Does Not Correlate With ET Induction or Cytokine Response

**Figure 4:**
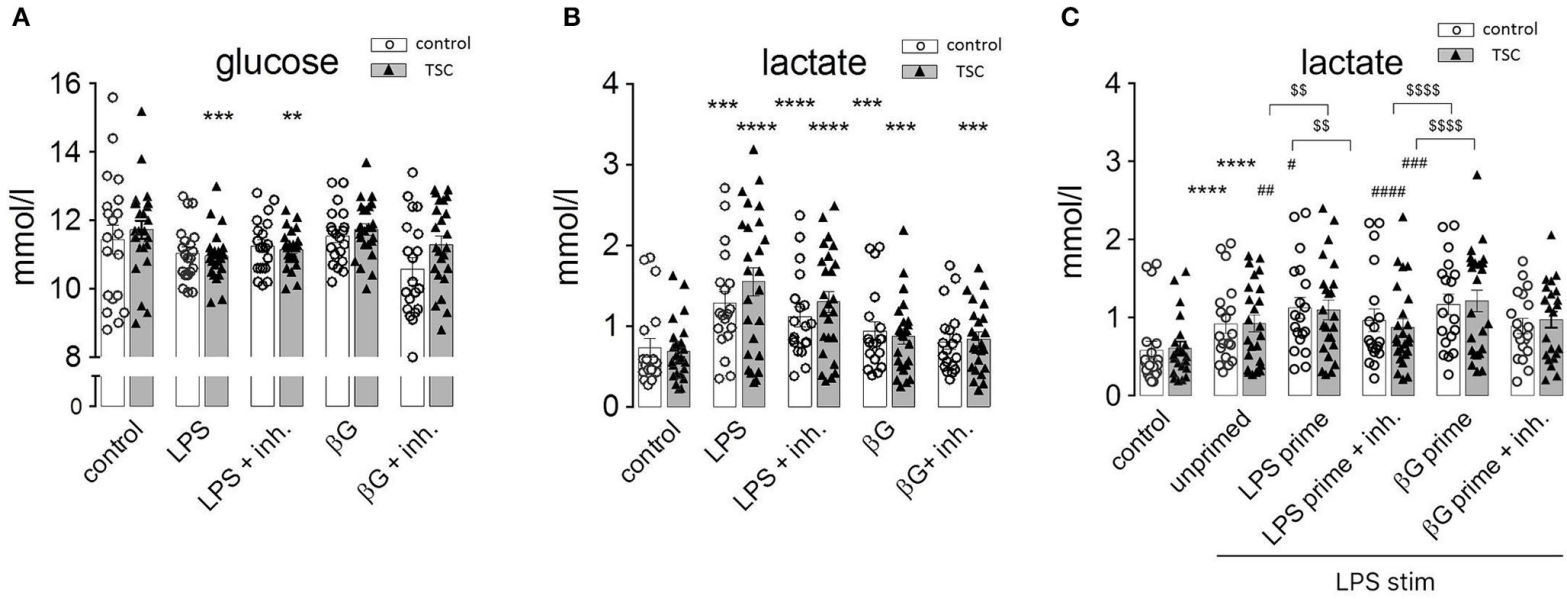
Pathogen-associated molecular pattern stimulation upregulates lactic fermentation, but this metabolic switch does not correlate or associate with endotoxin tolerance (ET). **(A)** Lactate levels in the supernatant of monocyte cultures from control donors and tuberous sclerosis (TSC) patients. The cells were pretreated as indicated with a mix of rapamycin and Torin (Inh) and challenged with lipopolysaccharide or ß-glucan (ßG). **(B)** Glucose levels in the supernatant of the same monocyte samples as in **(A)**. **(C)** Lactic acid production by monocytes from control and TSC patients subjected to the two-step priming/stimulation protocol for analysis of ET. White columns: healthy controls. Gray columns: TSC patients. Different significance symbols were used to mark different inter-group comparisons.

Monocytes exhibit dramatic metabolic rearrangements upon activation/stimulation with inflammatory agents (12, 14, 29). These changes supposedly represent a switch from aerobic mitochondrial respiration to anaerobic, glycolytic metabolism characterized by increased glucose consumption and lactic fermentation. Our own experiments were in line with this scenario as human monocytes exhibited a markedly and significantly enhanced release of lactate upon stimulation with LPS (Figure 4A). This was accompanied by higher glucose consumption, attaining a statistical significance for the TSC monocytes (Figure 4B). ß-Glucan exerted an analogous but somewhat weaker response than LPS. The TSC monocytes showed a trend toward higher lactate production than the control cells under LPS stimulation, whereas treatment with rapamycin/torin1 did not exert any marked effect on lactate levels. These data were consistent with the occurrence of a switch to lactic fermentation in LPS-stimulated monocytes. The inefficacy of rapamycin/Torin treatment in reverting the switch to aerobic glycolysis indicated that mTORC1 did not play a major role in this process.

In order to understand if this metabolic switch played a role in the induction of ET, we assessed lactate production by tolerant monocytes. As observed in Figure 4C, LPS-primed and re-stimulated (hence tolerant) monocytes exhibited an exacerbated lactate generation. Virtually the same effect was observed in ß-glucan-primed and restimulated (hence non-tolerant) monocytes. Thus, priming by LPS or ß-glucan induced an undistinguishable switch to lactic fermentation in tolerant and non-tolerant cells re-stimulated with LPS.

### ET Does Not Correlate With mTORC1 Activity and Is Not Mediated by Paracrine Signaling Mediators

**Figure 5:**
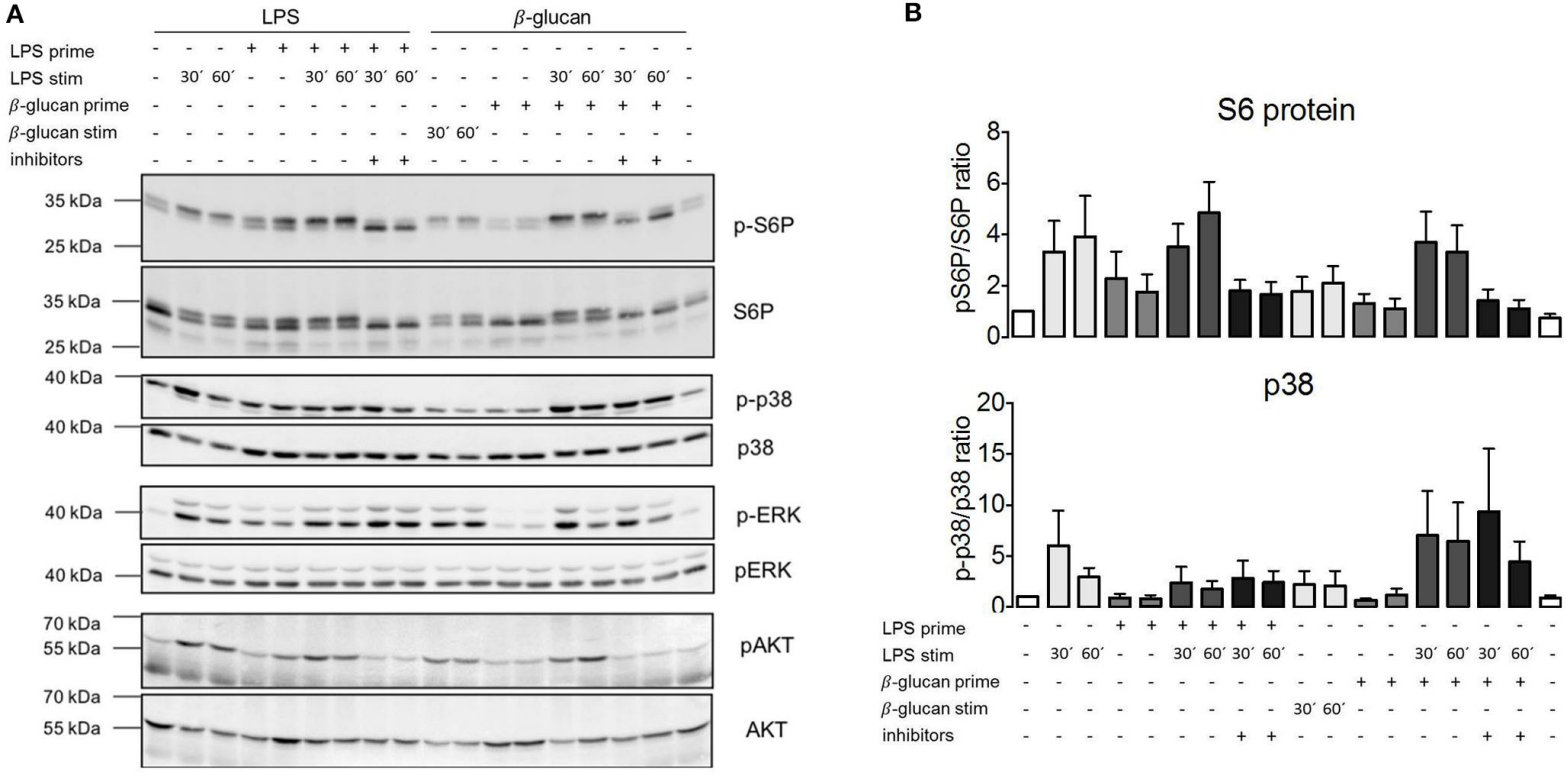
MTORC1 activation is not affected by endotoxin tolerance but lipopolysaccharide (LPS) fails to activate p38α in tolerant monocytes. **(A)** Monocytes from healthy donors were isolated and subjected to the indicated two-step stimulation protocols with LPS and or ß-glucan, including pretreatment with mTORC1 inhibitor mix as indicated. The cell extracts were processed for western blot against the indicated phosphorylated and total protein levels. The molecular size markers are indicated on the left side of the panels. **(B)** Bands for phosphorylated and total p38α and pS6 were quantified by densitometry, and the extent of activation was determined by plotting the ratio of phosphorylated/total protein. The quantification includes all measured samples (S6P: *n* = 6, p38α: *n* = 4) depicted as fold activation of the unstimulated samples. Data are presented as mean ± SEM.

The absence of the effects of TSC genotype or pharmacological mTORC1 inhibition on ET parameters prompted the question whether mTORC1 was activated following exposure to PAMPs under these experimental conditions. We took monocytes from control, healthy donors, and measured the phosphorylation of the mTORC1 downstream target S6-protein (S6P) by western blotting as a readout of pathway activation. As shown in Figure 5A, LPS activated mTORC1 as evidenced by the phosphorylation and concomitant mobility shift of S6P. This phosphorylation largely vanished after 24 h. Importantly, S6P was re-phosphorylated by a second LPS addition in tolerant cells (Figure 5A). The same pattern was observed for the phosphorylation/activation of Akt, an upstream activator of mTORC1. We concluded that the mTORC1 pathway was fully responsive to PAMP stimulation in the tolerant monocytes. Interestingly, activation of the parallel pro-inflammatory signaling pathway p38 was suppressed in the tolerized monocytes (Figure 5A), showing that ET had a distinct impact on the downstream transmission of the LPS signal to distinct pathways.

**Figure 6:**
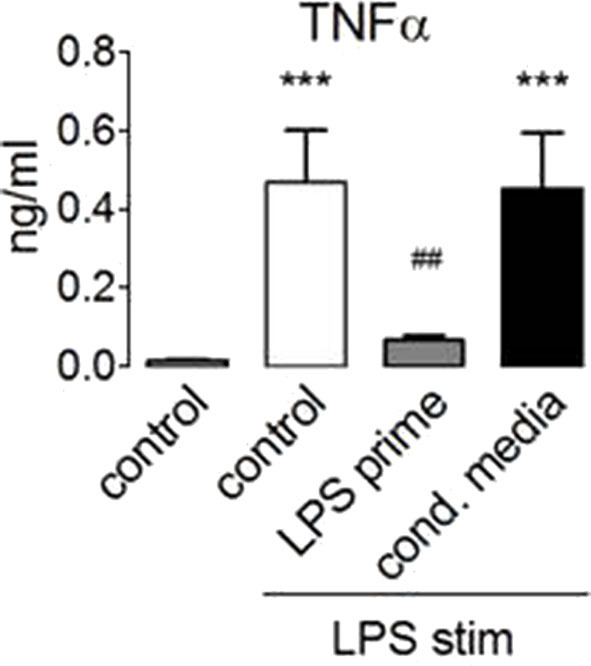
Paracrine factors do not mediate endotoxin tolerance induction. Monocytes from healthy donors were primed for 24 h with 100 ng/ml LPS or with conditioned medium obtained from monocytes 24 h after stimulation with 100 ng/ml LPS. The cells were re-stimulated with lipopolysaccharide, and TNFα production was assessed by ELISA. Different significance symbols were used to mark different inter-group comparisons.

These data showed that LPS activates mTORC1 and that mTORC1 activation by endotoxin proceeded normally in the tolerized monocytes. At the same time, mTORC1 activity is necessary for the production of IL-10 (30) (Figure 2A), an anti-inflammatory cytokine that has been linked before to the induction of ET (3). Since mTORC1 inhibition did not prevent ET, we reasoned that IL-10 or other paracrine mediators released in a mTORC1-dependent manner were unlikely to mediate the induction of ET in human monocytes. To test this assumption, we collected conditioned supernatant from human monocytes stimulated with LPS and used this medium to prime naïve monocytes prior to stimulation with LPS. As shown in Figure 6, LPS-elicited TNFα production was not compromised by the previous administration of a conditioned medium from tolerant monocytes, indicating that the paracrine factors released during priming are not crucially involved in the induction of ET.

## Discussion

mTORC1 coordinates resource availability and hormonal status with intracellular energy and nutrient expenditure and, as such, is predicted to be involved in processes involving re-wiring of metabolic pathways. Our experiments document an increase in lactic fermentation in human monocytes challenged with LPS or ß-glucan, which is in line with previous studies that reported Warburg-like metabolic reprogramming in activated macrophages (31). However, our data argue against a dominant role of mTORC1 in this process as the increase in lactic acid production was indistinguishable in control and TSC monocytes or in the presence of mTORC1 inhibition. While we cannot exclude that mTORC1 activity may be relevant for metabolic changes in monocytes under particular circumstances, e.g., in a background of distinct energy or nutrient availability, our findings illustrate that the PAMP-induced metabolic switch proceeds in the absence of a functional mTORC1 module. Similarly, we did not observe prominent effects of the TSC genotype, which causes high mTORC1 activity, on the cytokine response of TSC patient monocytes to LPS or ß-glucan besides a trend to mild overproduction of certain cytokines such as TNFα. Indeed the higher cytokine production in cells from TSC patients was blunted in those that had received everolimus treatment, pointing to a causal link between mTORC1 and the secretory activity of human monocytes, which is consistent with previous reports (32). Inversely, mTORC1 activity was critically required for the production of selected cytokines (IL-10, MIP1ß), the secretion of which dropped in a background of mTORC1 inhibition. Together with similar previous findings (30), this observation suggested that mTORC1 is differentially involved in the generation/secretion of distinct inflammatory cytokines. The reasons for this differential repercussion (considering that mTORC1 acts as a gatekeeper of global protein translation) are intriguing and could reflect a mechanism for adaptation of inflammatory cytokine release to the reigning nutrient/energy status. In this regard, we observed that monocytes from TSC patients showed enhanced LPS-induced IL-1ß production (see Figure 2). Since the activation of the inflammasome requires an initial priming step to stimulate the synthesis of caspase 1 and IL-1ß precursor proteins, we speculate that the intrinsically high mTORC1 activity of TSC cells likely boosts this priming reaction that precedes IL-1ß production and secretion. Interestingly, while we observed an inhibitory effect of chronic everolimus therapy on the secretory activity of TSC monocytes, e.g., for IL-1ß or TNFα (Figure 2B), acute mTORC1 inhibition did not cause the same effect. This evidenced that not every consequence of aberrantly high mTORC1 activity in TSC cells could be reversed by the acute inhibition of mTORC1, an observation that is not unprecedented (33). In this context, it is interesting to consider mTOR-independent effects of TSC loss in human monocytes. In particular, TSC1 acts as a co-chaperone of HSP90 (22, 34), and HSP90, in turn, reportedly modulates PAMP/TLR signaling at multiple levels, including the stabilization of functional TLR receptor complexes at the plasma membrane of human monocytes/macrophages (35). Taking all these findings together, we concluded that the effects of mTORC1 on monocyte cytokine secretion are multifaceted and impact differentially on individual cytokines. Moreover, the effects are most likely not mediated by mTORC1-dependent changes in cellular metabolism as TSC loss or mTOR inhibition had little impact on metabolic reprogramming in our experiments.

The monocytes exhibit a dramatic readjustment of their secretory and functional status upon entering a state of immune tolerance (3). Our data highlight that the hyperactivation of mTORC1 in TSC or its pharmacological inhibition did not preclude the induction of endotoxin tolerance in human monocytes, monitored here by the reduced or the altered endotoxin-induced production of inflammatory cytokines. These findings indicate that changes in mTORC1 activity are not involved in the induction of ET and have far-reaching implications. For example, it would argue against a role for endocrine loops involving cytokines whose secretion depends on mTORC1, at least in settings of *in vitro* ET induction. This includes, e.g., the anti-inflammatory cytokine IL-10, whose secretion is strictly contingent on mTORC1 (Figure 2). Our experiments using conditioned medium from primed human monocytes argue in the same direction as they excluded a contribution of extracellular factors in the induction of ET. Taken together, these considerations suggest that the mechanisms responsible for ET involve intracellular re-wiring processes that are largely independent of mTORC1. Since all presented data argue also against a role of metabolic reprogramming, the straightforward conclusion is that changes in the signaling machinery and/or genetic re-programming of the primed monocyte underlie the induction for ET. In this regard, we document that tolerant monocytes become unresponsive at the level of the p38 pathway, while other pathways (mTOR, Erk, and Akt) remain sensitive to LPS challenge. These findings suggest that uncoupling of p38 from TLR signaling could be one important feature of ET. Irrespective of the mechanism, this finding is consistent with the notion that tolerance leads to a qualitative change in LPS signaling, e.g., perhaps to a rearrangement of the proximal TLR4 signaling network in the tolerant monocyte. It will be intriguing to evaluate the functional consequences of defective p38 signaling in the stimulated monocyte and whether this can explain some of the features of tolerized cells.

In our experiments, we did not observe a significant training effect of the PAMP ß-glucan despite testing various sources of ß-glucan and different protocols. This confirms previous findings (23, 36) but contrasts with reports documenting an enhanced cytokine production in ß-glucan-primed monocytes (2, 24). We suspect that differences in the experimental protocols and normalization procedures underlie these different outcomes. Irrespective of these considerations, the results of ß-glucan stimulation shown herein are nevertheless intriguing as ß-glucan induces a comparably strong switch to lactic fermentation as LPS, in line with previous reports (12, 29). Moreover, ß-glucan stimulates the mTORC1 pathway as monitored at the level of S6-protein phosphorylation (Figure 5), to the same extent as LPS, yet ß-glucan does not induce ET, proving that stimulation of mTORC1 and metabolic re-wiring are not sufficient for the induction of ET. Thus, our findings provide strong evidence that mTORC1 activity and a metabolic switch to lactic fermentation are neither necessary nor sufficient for the induction of ET.

Taking this line of thinking one step further, it must be concluded that polarization of monocytes/macrophages [a strictly mTORC1-dependent process (26)] and tolerance induction (mTORC1-independent, as shown herein) are largely separate and independent processes. Indeed the relation between these two processes has been difficult to judge in the past because training or adaptation studies involved mostly experimental cytokine profiling, while polarization mostly relied on the assessment of marker signatures. Our data illustrate that ET does not depend on mTORC1 activity, which sets the adaptive process of ET clearly apart from the mTORC1-dependent program of monocyte/macrophage polarization.

ET represents a physiological adaptation process for shaping and adapting the inflammatory response to individual infection scenarios. However, unleashed or uncontrolled immune tolerance is thought to lie at the heart of critical conditions such as sepsis (5, 6). Sepsis is often accompanied and linked to metabolic comorbidities (insulin resistance, diabetes, obesity, and liver dysfunction) (5, 37), all of which do affect nutrient levels and nutrient/hormonal signaling in the critically ill patient. A better understanding of the role of mTORC1-dependent signaling in this context could help in devising new strategies of immune modulation in sepsis and other clinical settings. For another example, in solid organ transplantations, patients often receive mTORC1 inhibitors (everolimus and tacrolimus) as immunosuppressant. Our present findings, which show very limited consequences of TSC loss and/or mTORC1 inhibition on monocyte function and/or plasticity, suggest that immune suppression in these cases is most likely to result from a strong inhibition of adaptive immunity. Given the distinct contributions of innate vs. adaptive immune entities to the course of different syndromes and pathologies, it is tempting to consider mTORC1 inhibitors as a means to selectively modulate the immune response in different clinical settings in an individualized fashion.

In conclusion, while mTORC1 is a well-established player in the primary response of numerous immune cells [e.g., in T-cells (38) or monocytes, see Figure 1], our findings argue against a prominent contribution of mTORC1 to processes of immune cell adaptation, at least in monocytes. In line with this concept, TSC is not associated with a defective response to infection as judged by the absence of an increased incidence or severity of infectious episodes in TSC patients. Accordingly, we did not observe any conspicuous, unusually high incidence of infections or immune abnormalities in our TSC patient cohort, yet the clear impact of mTORC1 on monocyte polarization and the monocyte's secretory landscape [present data and (26)], along with its well-established function in T-cell activation and clonal expansion, underscore an important role of mTORC1 signaling in immune cell function and warrant further investigations to understand the role of metabolic mTORC1 signaling in the host response to infection.

## Data Availability Statement

The datasets generated for this study are available on request to the corresponding author.

## Ethics Statement

The studies involving human participants were reviewed and approved by Local ethics committee at the University Hospital Jena (Ethik-Kommission der Friedrich-Schiller Universität Jena an der Medizinischen Fakultät). Written informed consent to participate in this study was provided by the participants' legal guardian/next of kin.

## Author Contributions

KL designed and performed most of the experiments. RH organized and performed patient and healthy control donor recruitment and blood draw. RH and IR wrote ethics application and study protocol. IR designed the study and experiments and wrote the manuscript. All authors corrected and approved the manuscript.

## Conflict of Interest

The authors declare that the research was conducted in the absence of any commercial or financial relationships that could be construed as a potential conflict of interest.
